# A copy number variant scan in the autochthonous Valdostana Red Pied cattle breed and comparison with specialized dairy populations

**DOI:** 10.1371/journal.pone.0204669

**Published:** 2018-09-27

**Authors:** Maria Giuseppina Strillacci, Erica Gorla, Maria Cristina Cozzi, Mario Vevey, Francesca Genova, Kathy Scienski, Maria Longeri, Alessandro Bagnato

**Affiliations:** 1 Department of Veterinary Medicine, University of Milan, Milan, Italy; 2 Associazione Nazionale Allevatori Bovini Di Razza Valdostana, Gressan, Aosta, Italy; 3 Department of Animal Science, Texas A&M University, College Station, Texas, United States of America; China Agricultural University, CHINA

## Abstract

Copy number variants (CNVs) are an important source of genomic structural variation, recognized to influence phenotypic variation in many species. Many studies have focused on identifying CNVs within and between human and livestock populations alike, but only few have explored population-genetic properties in cattle based on CNVs derived from a high-density SNP array. We report a high-resolution CNV scan using Illumina’s 777k BovineHD Beadchip for Valdostana Red Pied (VRP), an autochthonous Italian dual-purpose cattle population reared in the Alps that did not undergo strong selection for production traits. After stringent quality control and filtering, CNVs were called across 108 bulls using the PennCNV software. A total of 6,784 CNVs were identified, summarized to 1,723 CNV regions (CNVRs) on 29 autosomes covering a total of ~59 Mb of the UMD3.1 assembly. Among the mapped CNVRs, there were 812 losses, 832 gains and 79 complexes. We subsequently performed a comparison of CNVs detected in the VRP and those available from published studies in the Italian Brown Swiss (IBS) and Mexican Holstein (HOL). A total of 171 CNVRs were common to all three breeds. Between VRP and IBS, 474 regions overlapped, while only 313 overlapped between VRP and HOL, indicating a more similar genetic background among populations with common origins, i.e. the Alps. The principal component, clustering and admixture analyses showed a clear separation of the three breeds into three distinct clusters. In order to describe the distribution of CNVs within and among breeds we used the pair V_ST_ statistic, considering only the CNVRs shared to more than 5 individuals (within breed). We identified unique and highly differentiated CNVs (n = 33), some of which could be due to specific breed selection and adaptation. Genes and QTL within these regions were characterized.

## Introduction

The use of genomic information in dairy cattle breeding has taken high priority in recent years, as genomic selection has been adopted to improve genetic gain for production traits such as milk production [1] and meat quality [2] in cattle breeding programs. In the last 50 years, artificial and natural selection has provoked changes within the cattle genome, causing relevant phenotypic and genetic variability and resulting in the adaptation to local environments [3].

Structural variations, as the Copy Number Variants (CNVs), are one of the major contributors to genetic diversity and phenotypic variation [4]. Liu et al., (2010) [5] underlined the importance of CNVs in disclosing genetic diversity among populations and in breeds evolution.

CNVs were defined as large-scale insertions and deletions, ranging from 50 bp to several megabases (Mb)[6]. Compared to SNPs, which are commonly used to detect the existing genetic variation in cattle, CNVs involve larger genomic regions and may have stronger effects on gene regulation and expression. These effects include the modification of gene dosage and structure, which in turn cause exposure of recessive alleles and the alteration of gene regulation [7,8]. Studies in several species have found that CNVs are sources of phenotypic variability as well as disease susceptibility, describing up to 30% of the genetic variation in gene expression [9,10].

CNVs have been mapped in several livestock species [11,12,13], although their use as markers to explain intra-breed genetic diversity has been explored in only a few species [14,3,15]. CNV properties used to explore the diversity and structure of cattle populations remains an issue of little investigation [16]. The study of genetic variation in local populations is a fundamental step in understanding the evolutionary processes that lead to the divergence and differentiation of breeds. Since the mid 20^th^ century, the strong selective pressure to increase milk production in cattle has led to the specialization of breeds that were once dual-purpose in the past (i.e. Brown Swiss) to where their structure in terms of size and physiology has drastically changed.

The Valdostana Red Pied (VRP), farmed in the Aosta Valley located in the northwest Alps of Italy, is an autochthonous dual-purpose cattle breed that did not undergo any specialized intensive selection for neither milk nor meat. This population is bred for milk and meat, and possesses fairly considerable milk production considering the size of the animal (mature weight of 500 kg on average). It is a well-adapted breed to harsh environments as those that animals face during summer pasture in the Alps. Therefore, it is thought that the VRP’s genetic background is a population that diverged less than specialized populations as the Brown, from the ancestral cattle populations of the Alps.

CNV diversity in cattle breeds may reveal the genetic basis of their respective phenotypic differences and provide insights on their adaptation to environments: extensive farming vs. intensive farming systems.

In this study we mapped the CNVs of 143 Valdostana Red Pied (VRP) bulls in order to identify structural variations in this breed’s genome. Additionally, we compare the VRP’s CNVs with those already identified in the Mexican Holstein (HOL) and Italian Brown Swiss (IBS) cattle to highlight genomic structure diversity possibly linked to differences in breed fitness. Breeds were chosen because of their selection histories. VRP remains a dual-purpose breed, HOL has been heavily selected for milk production and intensive farming and IBS, while a dairy cattle breed for not more than 20 years, was initially selected for dual-purpose characteristics.

## Results

### CNV and CNVR detection in VRP breed

The stringent quality control performed with SVS allowed for the identification of 35 outlier individuals that were identified according to the Derivative Log Ratio Spread (DLRS) and genomic wave factor values. A total of 6,784 CNVs were detected with PennCNV software across the 29 autosomal chromosomes in a final dataset of 108 VRP bulls. Among these, 3,990 were deletions (i.e. loss states 0 and 1) and 2,794 were duplications (i.e. gain states 3 and 4), with a deletions/duplications CNV ratio of 1.42 calculated as the total number of losses divided by the number of gains. The CNV count ranged from 38 to 141 CNVs per sample, with an average of 62 CNVs. Additionally, the average size of CNVs for an animal ranged from 31,558 to 103,139 bp, with an average value of 55,566 bp across all individuals. Table 1 shows the descriptive statistics of the identified CNVs (Total Copy Number according to [38]) and CNV regions (CNVRs) at population level according to their state.

**Table 1:** Descriptive statistics for CNVs and CNVRs detected in VRP breed.

Total Copy Number*	No.	Mean Length	Min Length	Max Length	Total Coverage
*CNVs*					
0	1,434	59,322	1,245	581,425	3.39%
1	2,556	45,839	1,264	523,180	5.72%
3	2,779	56,924	1,030	1,052,912	6.00%
4	15	52,381	3,270	273,013	0.01%
All	6,784	59,322	1,030	1,052,912	15.10%
*CNVRs*					
Loss	812	29,827.30	1,245	494,272	0.53%
Gain	832	26,438.23	1,030	692,847	0.88%
Complex	79	167,388.85	1,714	2,170,361	0.96%
All	1,723	34,498.03	1,030	2,170,361	2.36%

* 0 = deletion of two copies, 1 = deletion of one copy, 3 = single copy duplication, and 4 = double copy duplication

All the CNVs were merged into 1,723 unique CNVRs (832 gains, 812 losses and 79 complex) across all individuals, covering a total of 59.4 Mb of the genome, which corresponds to 2.36% of the bovine UMD3.1 assembly.

In S1 Table the complete list of CNVRs in the VPR is reported. The regions mapped in a large number of individuals were: chr12 at 72.42–74.59 Mb (n = 104 samples—loss) and 70.49–72.12 Mb (n = 91 samples—complex), chr5 at 117.28–117.64 Mb (n = 107 samples—gain), and chr10 at 23.89–25.26 Mb (n = 76 samples—gain). In some cases, subjects contribute with two or more adjacent CNVs to the location of these regions.

**Fig 1:**
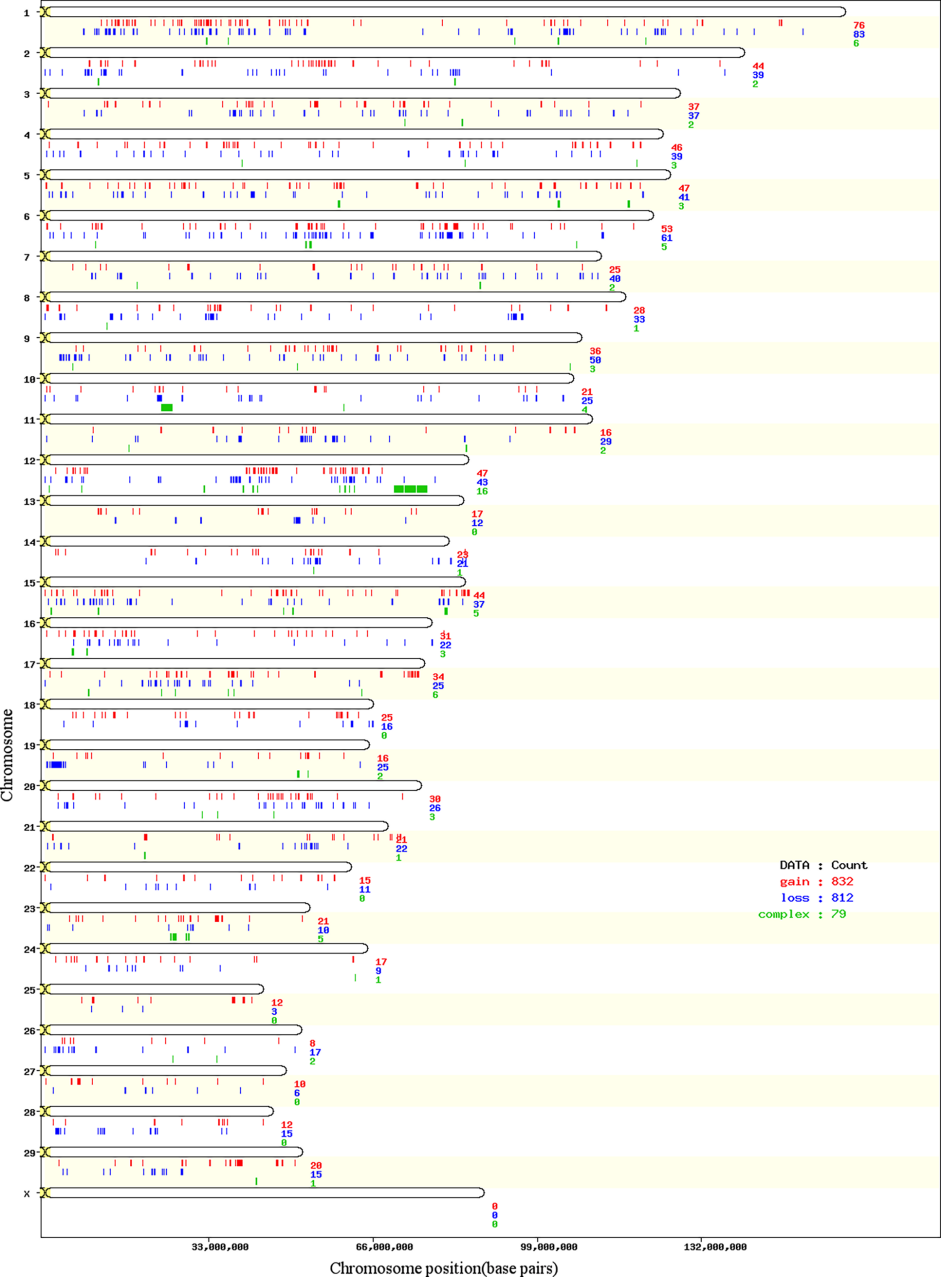
Distribution of the CNVRs on the chromosomes according to their state (gain, loss and complex).

In Fig 1, the map displays gain, loss and complex CNVRs on each chromosome.

S2 Table reports the number of CNVRs by chromosome and by state (gain, loss and complex) and the proportion of coverage by chromosome in the VPR. Although CNVRs were found on all autosomes, the number and the total size of CNVRs per chromosome were not correlated with their lengths.

**Fig 2:**
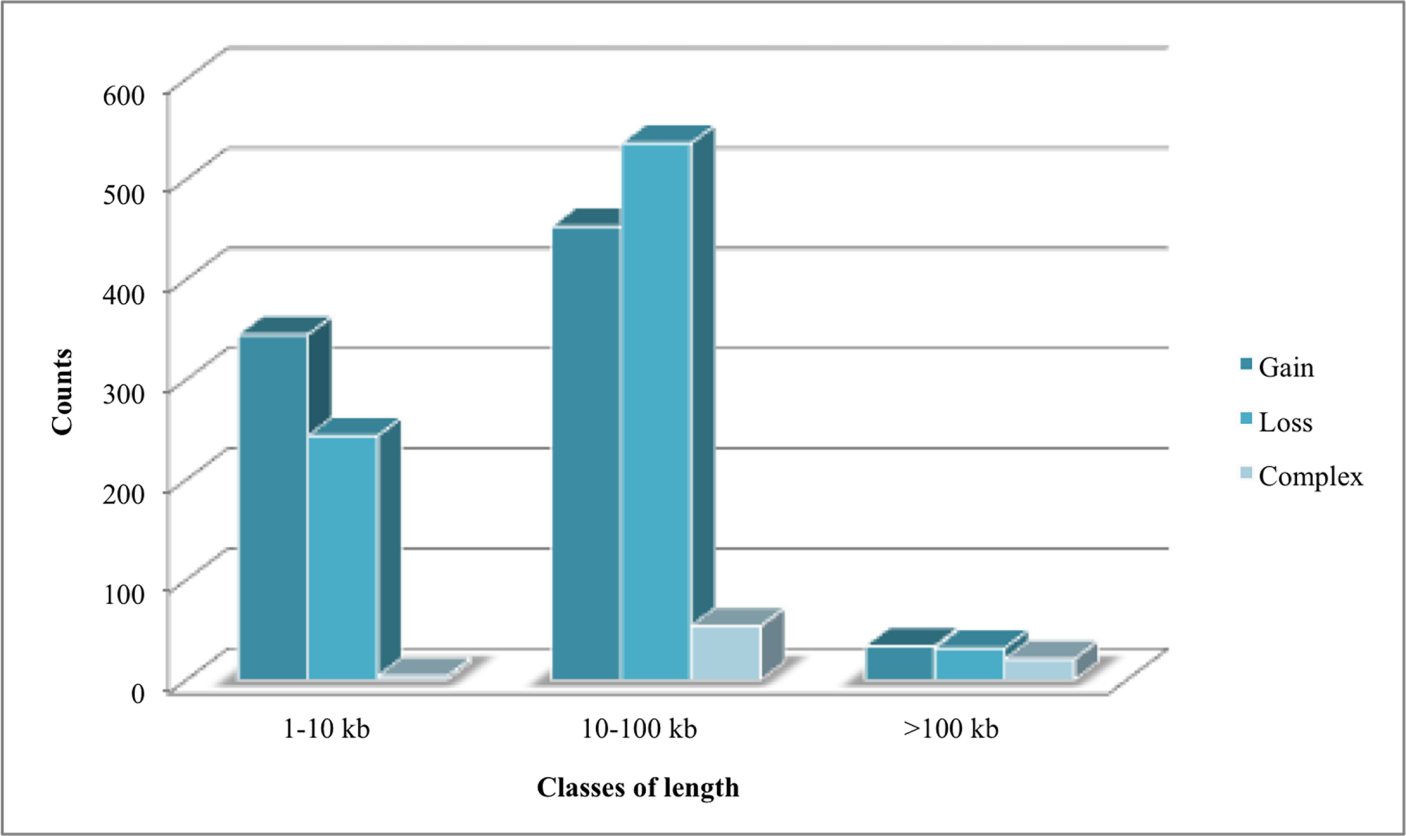
Distribution of CNVR lengths in VRP identified with PennCNV.

A classification based on CNVR length was performed for each state (i.e. gain, loss, complex) and the CNVRs have been divided into three classes of length: 1–10 kb, 10–100 kb, >100 kb (Fig 2). The majority of CNVRs identified in this study (n = 1,043) have a length comprised between 10 kb and 100 kb. The class of length comprised between 10 and 100 kb harbors the highest number of gain, loss and complex CNVRs. In addition, 593 CNVRs have a length comprised between 1 and 10 kb, while only 87 CNVRs had a size longer than 1 Mb.

**Fig 3:**
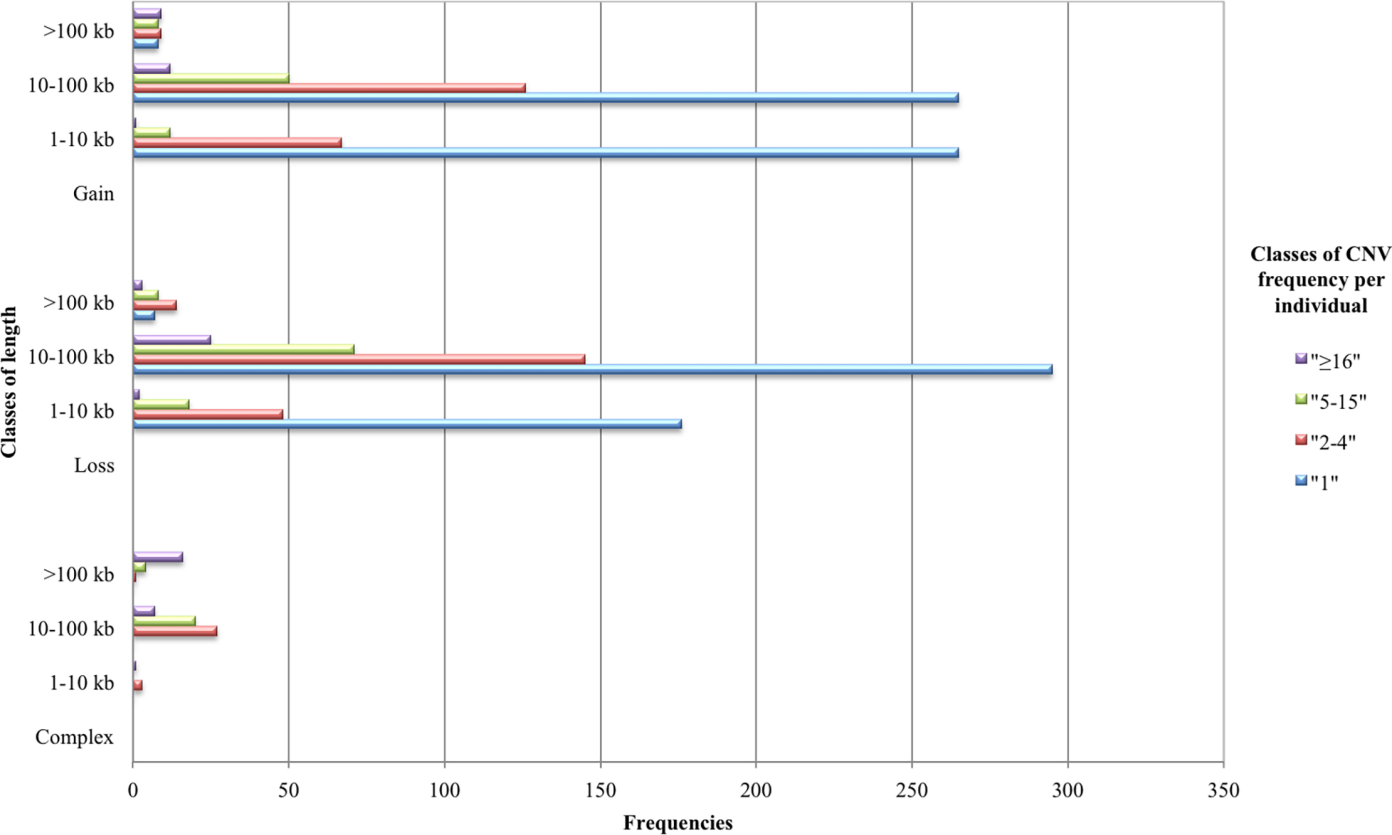
Sample count per individual class (1 singleton; 2–5; 5–15; >16) in each class of CNVR length (1–10; 10–100; >100 kb), according to CNVR states.

Additionally each class of CNVRs length has been divided into four classes of CNV frequency per individual (1, 2–4, 5–15, ≥ 16). The frequency count is shown in Fig 3. Thus, for every state, CNVRs were defined as singleton regions (if defined by one single individual), rare regions (if determined by 2–4 individuals), moderately recurring (if determined by 5–15 individuals), or recurring regions if including at least 16 individuals (Fig 3). In general, among the identified CNVRs, 1,061 (58.9%) were singleton, 440 (25.5%) were rare regions and, 267 (15.5%) are CNVRs identified in more than 5 individuals. If we consider CNVR states, the occurrences of singleton and rare regions were the most frequent both in gain and loss regions as shown in Fig 3.

#### Annotation of Valdostana Red Pied CNVRs

A total of 882 Ensembl gene IDs (Ensembl UMD3.1), corresponding to 442 genes with an official ID, have been identified in the 1,723 CNVRs of the VPR. Five hundred and thirty-six regions (31.1%) encompassed one or more genes, while 1,187 (68.9%) did not involve any gene (S3 Table).

The GO Term and KEGG pathway analysis was performed using the DAVID Classification database. After FDR (p-value < 0.05), terms resulting as statistically significant included 12 genes involved in heart development as “Biological Process,” and 4 genes involved in glucoside activity as “Molecular Component.” The complete list of Biological Process, Cellular Component, and Molecular Function is reported in S4 Table.

### Comparison of CNVs across populations

A comparison among VRP, HOL, and IBS cattle breeds was performed using CNVs called here and previously published, summarized in S5 Table and in the Venn diagram of S1 Fig. We observe that 171 CNVRs are shared among the three breeds, while 1,107, 1,800, and 1,161 unique CNVRs belong to the VRP, IBS and HOL, respectively. In particular, the CNVRs found in HOL overlap with 18.16% (313 CNVRs) of those found in VRP, while the identified regions in IBS overlap with 27.51% (474 CNVRs) of those found in VRP. Considering the lengths of the common 171 CNVRs, we can observe that those shared by VRP and IBS have an average length of 29.82 Mb (50.17% of the length of the CNVRs identified in this study), while the ones common to HOL and VRP, show an average length of 24.15 Mb (40.06% of the length of the CNVRs detected).

#### Principal component analysis

**Fig 4:**
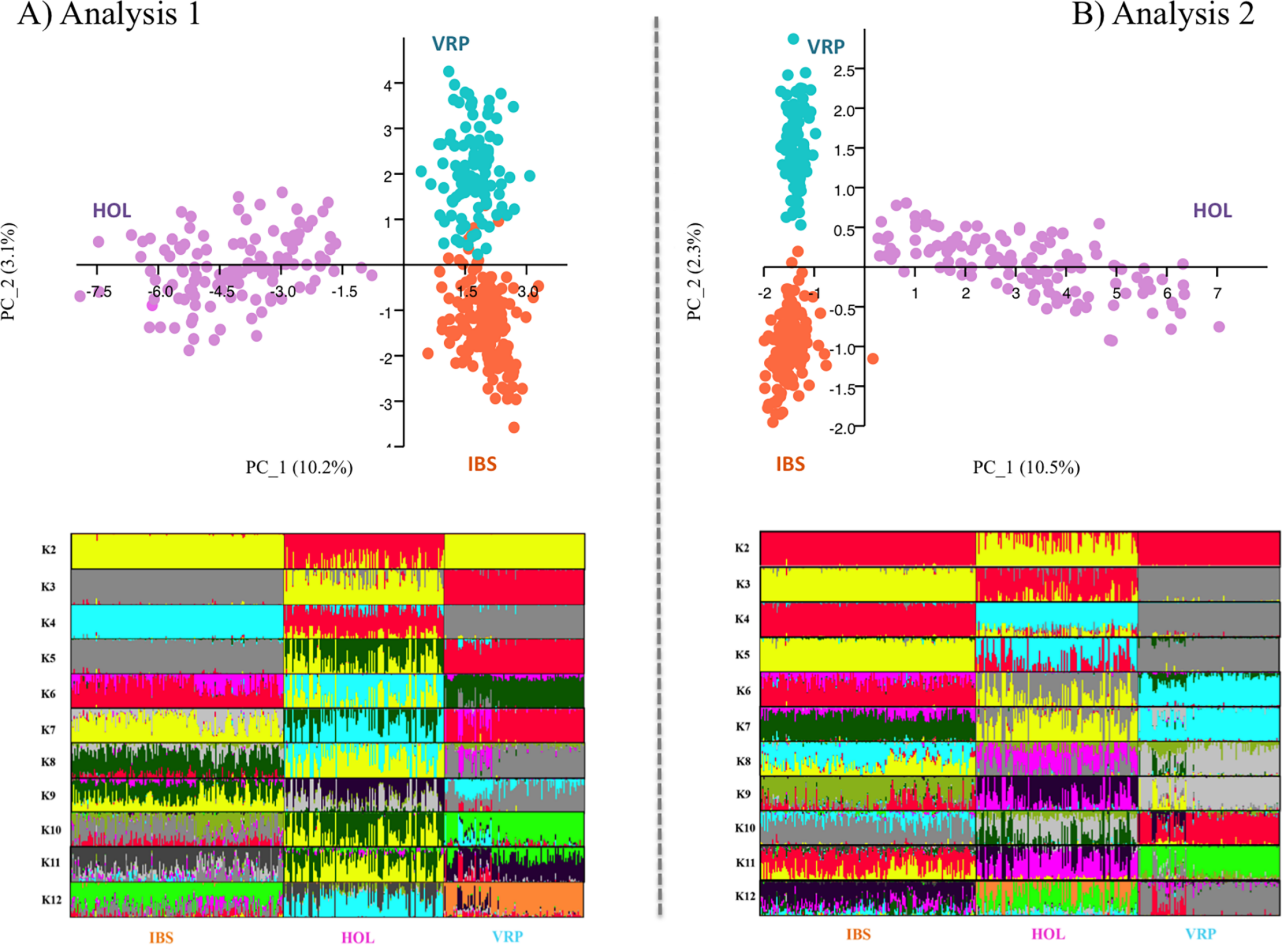
PCAs and population STRUCTURE analyses of three cattle breeds (VRP, IBS and HOL) based on CNVs. Twelve subpopulation clusters inferred by STRUCTURE are represented by different colors (K2-K12). A) Analyses run considering presence or absence of a CNV in a CNVR as described as Analysis 1 in the Materials and Methods section. B) Analyses run considering the CNV total copy number in a CNVR as described as Analysis 2 in the Materials and Methods section.

The first two principal components of the PCAs shown in Fig 4A explain 10.2% and 3.1% respectively of the total variability of data (PC1 and PC2). The same occurs for PCA in Fig 4B where 10.5% and 2.3% of the total genetic variation is explained by PC1 and PC2. Both analyses clearly identified three clusters corresponding to the three breeds (Fig 4A and 4B). While VRP and IBS breeds appeared to be closer, a clear separation resulted between IBS and VRP in respect to HOL.

#### Clustering to infer population structure

The STRUCTURE software was employed to analyze the genetic structure of the 396 animals of IBS, VRP and HOL. The analysis identified the true number of clusters (subpopulation) in which it is possible to divide the considered pools of individuals. i.e. VRP, IBS and HOL. Both the analyses (Analyses 1 and 2) assumed a model with 12 clusters (K = 12). Based on the heuristic test, the estimated likelihood (LnP (D)) values were used to obtain the ΔK values in order to distinguish the break in slope of the distribution of LnP (D) values at the true K. The analyses identify K = 3 as the likely K value suggesting that the population should be divided into 3 genetic groups: the VPR, the IBS and the HOL. In both analyses at K = 2, VRP and IBS were clearly assigned to a unique group distinct from HOL. At K = 3, the three breeds resulted in a clear separation of three clusters and most of the individuals were assigned to a cluster according to the breed division. From K = 4 to K = 12, the high level of admixture in each of the breeds (in particular in the HOL) shows that the successive clustering is not significant (Fig 4A and 4B).

**Fig 5:**
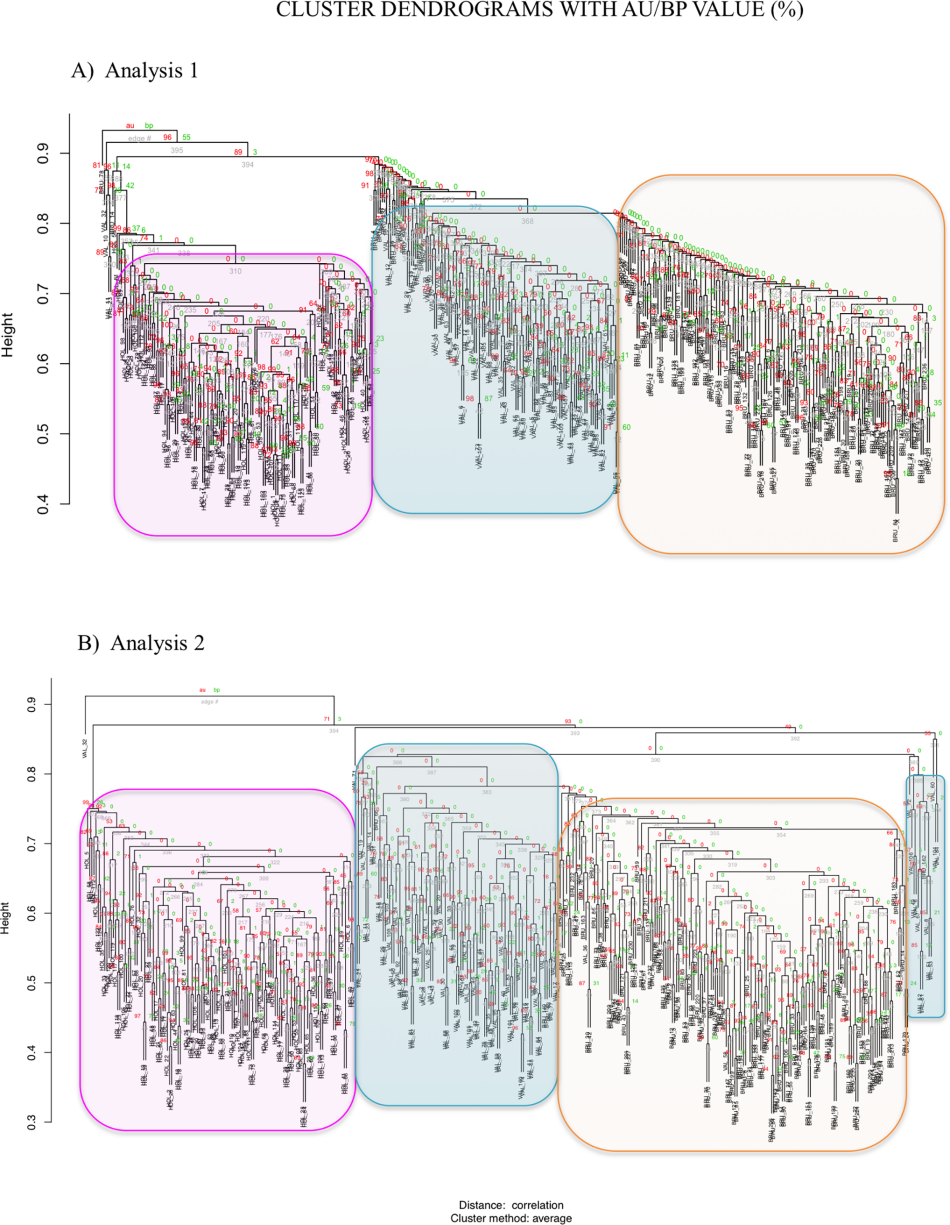
Dendrograms obtained from clustering analysis based on common CNVRs of VRP, IBS and HOL breeds.

The cluster trees represented in Fig 5A and 5B were built using the CNVR differences identified in the three considered populations. Each node of the trees reports the AU-P and Bootstrap probability values and the edge number. As reported by [17] the AU-P value is considered more accurate than the BP-P value. Even if many AU-P values reported for every node of the tree are low, maybe due to the number of CNVRs considered in this analysis (171 regions share among the three breeds), the majority of individuals are grouped in three distinct clusters corresponding to the three populations (breed-cluster). To be noted that, IBS and VRP, although separated in different clusters, come from a common node.

#### Population Differentiated CNVs on V_ST_

In order to test if the CNVs can be related with population-specific selection, we calculated the pairwise V_ST_ among every combination of the three breeds (HOL vs IBS, VRP vs HOL, and VRP vs IBS). The V_ST_ statistic defines values that range from 0 to 1; the high V_ST_ values (close to 1), similar to F_ST_, suggest differentiation between populations, while low values (close to 0) are indicative of very similar populations.

**Fig 6:**
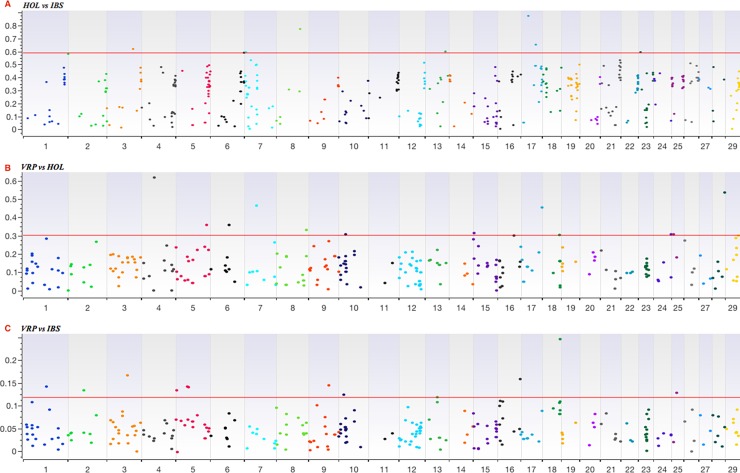
Genome wide V_ST_ value plots for CNVs in the combinations: A) HOL vs IBS; B) VRP vs HOL; C) VRP vs IBS.

To calculate the V_ST_ we used a total of 930 CNVs (only those identified in at least 5 individuals in each population), defined by 1,222 SNPs. The defined threshold, taking into account the pairwise of V_ST_ identified a total of 33 CNVs (Fig 6): 8 for HOL vs IBS; 13 for VPR vs HOL; 12 for VPR vs IBS. The genes and QTL annotated in these CNVs are reported in Table 2.

**Table 2:** List of CNVRs and gene and QTL annotation for pairwise V_ST_ > Mean + 2 S.D.

CHR	CNV Start	CNV End	Length	V_ST_	IND*	Genes			QTL**
**VPR *vs* IBS**									
1	83218713	83238102	19389	0.141	5	*EPHB3*			Conformation score QTL (106404, 106405), Average daily gain QTL (106246), Muscularity QTL (106247, 106248)
2	56375294	56403140	27846	0.132	5				
3	71477185	71486626	9441	0.165	11				
5	3434356	3439861	5505	0.133	6				
5	40181727	40209934	28207	0.141	6	*CNTN1*			
5	44705963	44718715	12752	0.14	5	*LYZ*			
9	71525299	71608476	83177	0.143	7				
10	17775153	17784123	8970	0.123	16	*LRRC49*			
13	43884430	43940108	55678	0.117	21	*AKR1C3*			
16	7901886	7948314	46428	0.11	12				
16	80271680	80284738	13058	0.157	7				
18	61894649	61918012	23363	0.246	37				
25	18666885	18674448	7563	0.128	11	*ERI2*, *REXO5*, *DCUN1D3*			
**HOL *vs* IBS**									
3	93310320	93315045	4725	0.615	7				Somatic cell score QTL (122082)
6	118543527	118545281	1754	0.587	5	*SORCS2*			
7	4226753	4238450	11697	0.591	7	*COPE*			
8	83242450	83261773	19323	0.769	5	*TSPY*			
13	70667271	70698983	31712	0.6	21	*LPIN3*, *EMILIN3*			
17	25056695	25119996	63301	0.874	97	*PRAME*			Average daily gain QTL (106236), Conformation score QTL (106238, 106239)
17	51115979	51370688	254709	0.651	60				Conformation score QTL (106240)
23	7655804	7688981	33177	0.595	58	*BAK1*, *GGNBP1*, *ITPR3*			
**VRP *vs* HOL**									
4	45062559	45072215	9656	0.618	6	*RELN*			
5	108810406	108866833	56427	0.358	6	*DCP1B*			
6	66451170	66465621	14451	0.358	5	*GABRA2*			
7	43487164	43498441	11277	0.462	67	*LOC788287*			Calving ease (maternal) QTL (106493)
8	105250028	105303832	53804	0.331	7	*COL27A1*			
10	23133923	23160598	26675	0.305	16				
15	1277543	1312041	34498	0.312	27				
16	56458959	56475433	16474	0.3	26	*SLC9C2*			
17	73004371	73023888	19517	0.453	7	*ZNF280B*, *ZNF280A*			
18	59154291	59182962	28671	0.301	5				Length of productive life QTL (123783)
24	61918390	62143246	224856	0.304	9	*BCL2*, *KDSR*			Body weight gain QTL (69320), Daughter pregnancy rate QTL (107040)
25	7380550	7388001	7451	0.307	6				Lean meat yield QTL (36946)
28	43916806	43924903	8097	0.534	7				

*IND = individuals per CNVR

** https://www.animalgenome.org/cgi-bin/QTLdb/BT/index

## Discussion

Although recent studies on CNVs in cattle breeds using high-density SNP chips have been performed, limited knowledge regarding genetic variability and CNV characterization in local populations like the VRP is available. This study is the first CNV scan on the VRP using a high-density SNP chip, and provides valuable information of the structural genomic variation able to enrich the Bovine CNV map. A total of 6,784 CNVs were detected in the autosomes of 108 VRP bulls, and breed-specific regions were identified comparing CNVs mapped here and those available from previously published studies for IBS (n = 164) [18] and HOL (n = 124) [19] populations. We observed a similar number of duplications (gain state) and deletions (loss state) in VRP and IBS, while the number of deletions (loss state) is superior to the number of duplications (gain state) in the HOL breed. The latter result was previously reported for the Holstein breed in several studies based on SNPs [20] and whole genome sequencing [21]. These results suggest the existence of high genetic variability among these breeds.

When we assessed population structure, both principal component analyses revealed that the three cattle breeds form non-overlapping clusters, which is evident given that they are three separated populations, even though the second PCA shows a clearer separation among IBS and VRP. The same results are found by the hierarchical clustering, which also exhibits that the HOL, VRP and IBS samples are grouped in three distinct clusters. The AU/BP values express the probability that the subdivision for that specific note truly exists: lover values are expressing, in this case, that the two individuals are similar in terms of CNV composition. As the CNV are not as frequent in the genome as the SNP, they provide a more limited input information. The CNV information shows anyhow that is able to provide a good information for separating the population among them.

Also, both the admixture analyses revealed that at K = 3 the three breeds result in three clearly separated clusters, and most of the individuals are assigned to a cluster according to their breed division. Very interestingly at K = 2 IBS and VPR result a unique genetic population. These two populations are autochthonous Alpine populations and possibly share a common genetic background. In addition, since the implementation of the selection programs in Italy occurring in early 1960s till 1990s (i.e. for 30 years), both VPR and IBS were also sharing a similar selection criterion: milk yield, meat production and especially adaptation to pasture. This latter characteristic is fundamental for breeds that during summer face the environmental challenge of pasturing in harsh mountain. This is still the ongoing selection objective for the VPR, while the IBS selection pushed in the last 30 years towards the specialization of the population as a dairy breed. Nowadays, in fact, the IBS is a specialized dairy breed with a large proportion of genes coming from the US Brown, historically selected for milk production. The results of this study show that IBS and VPR still are very close populations as the 30 years of strong directional selection in the IBS is still not sufficient to completely differentiate the two populations.

Regarding the HOL since 1950, Mexico has imported Holstein germplasm (mainly animals and semen) largely from the USA and Canada to increase the productivity of its dairy cattle populations [22]. The same occurred in Italian Holstein where more than 80% of the genetic origin is attributed to US bulls [23]. The HOL population here analyzed thus can be considered a representative sample of the genetic background that USA population has diffused all over the world in the last century after importation from the Holstein and Frisian regions of north Europe. The HOL population then has an origin mostly completely different than VPR and IBS. This results clear at K = 2 where HOL population is clustered separately from VPR and IBS. Additionally the HOL at K = 3 is showing common CNV regions with the IBS and in a very minor extent to the VPR. We may speculate that this has occurred because the selection in the IBS to increase milk production and the introgression of Brown Swiss bulls from USA, already strongly selected for milk production, has generated CNVs of common importance between HOL and IBS. Nevertheless at K = 3 IBS and VPR remain very well differentiated from the HOL and results to be 2 distinct populations.

The pairwise V_ST_ for the three comparisons (HOL vs IBS, VRP vs HOL, and VRP vs IBS) was estimated in order to identify CNVs under a population-specific selection. According to the V_ST_, we identified a total of 33 CNVs that differing in frequencies in the above-mentioned comparisons, 8, 12 and 13, respectively, could be considered involved in breed selection. The high V_ST_ values in the comparison of VRP vs IBS, as shown in Table 2, are closer to zero in respect to the V_ST_ results obtained comparing HOL to the other two breeds, which are closer to one. This confirms the genetic similarity described above between the two populations and their difference from the HOL.

Among the 33 genomic regions, 21 CNVs encompass 22 genes, some of which have a well-known phenotype associated in cattle or in other species. The lysozyme gene (*LYZ*) (VPR vs IBS) on BTA 5, encodes for the 1,4-beta-N-acetylmuramidase C. It belongs to a class of enzymes that lyse the cell walls of certain gram-positive bacteria and has also been described in other important functions including inactivation of certain viruses, enhancement of phagocytic activity for leukocytes and macrophages, and control of inflammation [24]. The CNVR in the VRP is a gain, while is neutral in the IBS [19]. We may speculate that the *LYZ* gene in this CNVR may play a role in the immune response to bacterial infections as e.g. the mastitis. VPR is recognized by farmers as particularly capable to coop with mastitis infection. A recognized indicator of mastitis occurrence is the Somatic Cell Score (SCS), a log-linear transformation of the somatic cell count in milk (among them leucocytes) increasing when mastitis is occurring [25]. Average population SCS are reported by [26] for the IBS 3.45 (s.d. 2) and by [27] 2.65 (s.d. 2.09) for the VRP providing an indication of the difference in mastitis resentence between these breeds. For the same breed comparison, the CNVR on BTA 10 contains leucine-rich repeat containing 49 (*LRRC49*), which has been associated with subcutaneous fat and marbling score in the Canchim beef breed by [28]. The VPR is a double purpose cattle breed with specific directional selection for meat production and meat quality, showing a gain in this CNVR region differently from the neutral state in the IBS. In the IBS the directional selection for milk yield ongoing for 30 years, caused a drastic change in animal body structure (and its physiology) from a double purpose one to a very skinny structure (see additional Fig 2).

The comparison of VRP vs HOL showed as a possible candidate gene under differential directional selection the reelin (*RELN)* gene located in a CNVR on BTA 4, a loss in VRP and a gain in HOL [18]. As reported by [29] *RELN* is involved in the regulation of mammary gland morphogenesis and resulted downregulated in lactating pregnant cows, showing an imbalance and then the possible lower availability of this protein affecting embryo differentiation and development. Lastly, the *SLC9C2* gene is located within a CNVR on BTA4 (gain in VPR, neutral in HOL [18]) and is associated with a polyunsaturated fatty acid profile in intramuscular fat of the *Longissimus thoracis* muscle in a Nellore cattle population (Lemos, 2017. Online Thesis; http://hdl.handle.net/11449/150817). We can again speculate that this reflect the characteristics of the two populations, the VPR double purpose selected also for meat quality, and the HOL always selected to deliver energy just to milk production and not to develop muscular mass.

In respect to the HOL vs IBS comparison, the BCL2 antagonist/killer 1 (*BAK1*), located in a CNVR on BTA 23, has been associated with carcass measurements in beef cattle breeds [30]. The sortilin related VPS10 domain containing receptor 2 (*SORCS*2) in a CNVR on BTA 6 has been related to lipid metabolism in different mammal species and specifically with backfat thickness in the Nellore beef breed [31]. Finally, the lipin 3 (*LPIN3*) is in a CNVR on BTA 13 has been linked to both lipodystrophy in humans and backfat thickness in cattle by [32] and identified as a potential marker for hepatic metabolic adaptations to negative energy balance, as well as for altered physiological state occurring during the transition period in cattle, like adipose tissue lipolysis or hepatic fatty acid oxidation by [33]. All these three genes are located in CNVRs with a loss for HOL and a neutral state for IBS. Interestingly the same occurs for VRP. These findings are a suggestion that the strong directional selection occurring in the Holstein populations, since the application of breeding programs in this breed, may have generated the loss occurring in the three CNVR. The diminished capacity to efficiently build energy reserves, as a consequence of the selection occurred, is well known [34] as the decreased ability to coop with metabolic health during the transition period [34, 35].

In a more general context *EPHB3*, *PRAME*, *TSPY*, *and ZNF280B* were identified by [16] as genes under selection in a comparison between Taurine cattle (included Holstein and Brown Swiss cattle breeds) and two African multipurpose populations using V_ST_. Finally, 12 QTLs overlapped with the significant CNVRs resulting from the V_ST_ analysis, and some of them have already been associated to functional processes in cattle (Table 2).

In general, our analyses revealed distinctiveness among the IBS and VRP in respect to HOL, especially related to genes regulating the distribution of intramuscular lipids, which is indicating a difference in metabolism of individuals. In particular we may speculate that the use of resources in HOL is not addressed to fat deposition and in a more general context to body weight, differently than in the double purpose VPR breed, an in a minor extent in the IBS, a double purpose breed till 30 years ago.

## Conclusions

In this project, we performed the first CNV mapping in an autochthonous cattle population, the Valdostana Red Pied breed, using high-density SNP genotypes. The study permitted to disclose a CNV map in a local population well adapted to a harsh environment., and to compare it with 2 cosmopolitan populations, the Holstein and the Brown Swiss. One of the major indication of this study is that the directional selection occurring in population is affecting the genome in term of CNVs. Particularly the comparison among a very selected and specialized population, the HOL, a population as the Italian Brown Swiss where a directional selection occurred only recently, and a population under a very limited selection pressure for milk and meat but maintained adapted to environment as the VPR, discloses differentiated CNVRs where genes and QTL related to their selection history are annotated.

## Materials and methods

### Sampling and genotyping

The Associazione Nazionale Allevatori Bovini di Razza Valdostana (A.N.A.Bo.Ra.Va.) provided commercial semen doses of 143 bulls. No animals were involved directly in this study; consequently, no ethical approval was required.

Genomic DNA was extracted from semen using the ZR Genomic DNA TM Tissue MiniPrep (Zymo, Irvine, CA, U.S.A.). DNA was quantified using NanoQuant Infinite-m200 (Tecan, Männedorf, Switzerland) and diluted to 50 ng/μl as required in order to apply the Illumina Infinium protocol. DNA samples were genotyped using BovineHD Genotyping BeadChip Illumina (Illumina Inc., San Diego, USA) containing 777,962 polymorphic SNPs with a median <3 kb gap spacing.

### CNV and CNVR detection in VRP breed

Intensity signals from all SNPs were clustered using the Illumina BeadStudio software V.2.0 (Illumina Inc.). Samples with a call rate below 98% were excluded. The signal intensity data of log-R ratio (LRR) and B allele frequency (BAF) were exported from the Illumina BeadStudio software on all the autosomes. As quality control, the overall distribution of derivative log ratio spread (DLRS) values was used in the SVS 8.4 software (Golden Helix Inc.) to identify and filter outlier samples [36]. In addition, individuals were screened for their GC content, which is correlated to a long range waviness of LogR ratio values. Outlier samples were detected by the SVS 8.4 for waviness [37] and those identified were deleted.

The PennCNV software [38] (http://penncnv.openbioinformatics.org/en/latest/) was used for CNV calling in the VRP breed. PennCNV is based on a Hidden Markov Model (HMM) algorithm using as input the LRR and BAF data from the SNP arrays. Only samples with a standard deviation (SD) of LRR <0.30 and with default set of BAF drift as 0.01 were used to call CNV. Additionally, a minimum of three adjacent SNPs was required for the detection. The CNV regions (CNVRs) were defined as described by [39], using the BedTools software (-mergeBed command) [40], through merging overlapping CNVs by at least 1 bp. CNVRs were classified as “gain” if there was a duplication of the genome, “loss” if there was a deletion, or “complex” if the region comprised both gain and loss events.

### Comparison of CNVs across populations

In this study, we used CNVs to study the population-genetic properties in cattle. In order to identify genomic diversity among the three populations (VPR, HOL, and IBS), we used the individual CNVs available from [18] and those identified in Italian bulls selected from [19] all of them identified from individuals genotyped with the same Illumina’s 777K BovineHD Beadchip. CNV calling was performed following the same procedures as in our study, and only CNVs identified (within each breed) in at least five individuals were considered in this comparison.

Based on CNVs, two different matrices (number of individuals by number of CNVs) were built and applied for analyzing population genetic properties. The first matrix was build by presence (“1”) or absence (“0”) of a CNV in a CNVR, without considering if CNVs were a gain or a loss (Analysis 1) as used in the studies of [13–15]. The second matrix was built according to the CNV total copy number: “0” deletion of two copies, “1” deletion of one copy, “2” normal state (absence of CNV in that region), “3” single copy duplication and “4” double copy duplication (Analysis 2) as applied in [41]. The use of two different approaches to inform the matrices built was chosen to explore if the presence of the CNV in a CNVR is sufficient to discriminate genomic variation among individuals and if the availability of the CNV genotype is providing additional information.

Different approaches and software were used in order to disclose population structure and diversification of the three breeds considered. The Past software [42] was employed to perform two different principal component analyses (PCAs) of pairwise individual genetic distances based on CNV frequencies in CNVRs classified according to Analyses 1 and Analysis 2 (as above). The STRUCTURE v2.3.4 software [43, 44] was used to obtain a complete representation of the population structure of the considered breeds, using both the two matrices built as hereinbefore described. The Admixture model of STRUCTURE without the LocPrior option was used, with a 5,000 burning period and 10,000 iterations, performing five repeats for each K value from 2 to 12 and assuming three different populations. On the basis of STRUCTURE results, the best K values were calculated using the Structure Harvester software [45], which provides the DeltaK values according to the heuristic method reported by [46]. The Distruct software [47] was utilized to graphically visualize each cluster assignment for K of 2 to 12. A clustering analysis was then performed using the pvclust package of the software R [17], applying a hierarchical agglomerative clustering to the scoring matrices based on Analysis 1 and Analysis 2 (as above). In order to obtain the Approximately Unbiased P-value (AU) and identify the branches robustness, a multiscale bootstrap resampling (n = 10,000 bootstraps) was used. For the hierarchical clustering method, we employed the Unweighted Pair Group Method with Arithmetic mean (UPGMA).

In order to identify novel and exclusive population-differentiated loci, the V_ST_ statistic (highly correlated with Wright’s fixation index of F_ST_) was used. As defined in [39], V_ST_ is calculated at SNP level by considering (V_T_-V_S_)/V_T_, where V_T_ is the variance in LRRs of SNPs (within defined CNVR) estimated among individuals of two populations and V_S_ is the average variance within each breed, weighted for breed size (in our case: VRP *vs* HOL, VPR *vs* IBS, and HOL *vs* IBS). SNP V_ST_ values in a given CNVR have been averaged to obtain a mean V_ST_ value for each CNVR. Significance threshold was set to the nominal V_ST_ value corresponding to the top 5%.

### Annotation and Gene Ontology and Pathway Analysis

The full Ensembl UMD3.1 gene set for the autosomal chromosomes was downloaded from Ensemble Genome Browse database (release 90—August 2017), using BioMart (http://www.ensembl.org/biomart). Gene ontology (GO) and KEGG pathways analyses were performed with the high classification stringency option and FDR correction, using the DAVID database (https://david.ncifcrf.gov). The analyses allowed the identification of molecular functions, biological processes, cellular components and pathways for the genes included in the consensus CNVRs. In addition, the National Animal Genome Research Program database (https://www.animalgenome.org) was utilized to catalogue bovine QTL overlapping in both VRP’s CNVRs and within significant CNVs.

## Supporting information

S1 Fig Venn Diagram and descriptive statistic summary representing common and unique CNVRs found among the three populations considered. (PDF)

S1 Table Valdostana Red Pied CNVRs. (XLSX)

S2 Table Descriptive statistics of VRP CNVRs classified according to state (gain, loss and complex) by chromosome (Chr). (DOCX)

S3 Table List of VRP CNVRs and annotated genes as identified by the UMD3.1 assembly. (XLSX)

S4 Table GO Term and Pathway Analysis in VRP performed using DAVID on line database. Sheet 1: Genes clustered; Sheet 2: Genes not clustered.
(XLSX)

S5 Table Descriptive statistics of CNV and CNVR counts identified with PennCNV in Valdostana Red Pied (VRP), Holstein (HOL) and Italian Brown Swiss (IBS). (DOCX)
