# Supplementary figures and images for: A copy number variant scan in the autochthonous Valdostana Red Pied cattle breed and comparison with specialized dairy populations
Source: PLoS One. 2018 Sep 27;13(9):e0204669. doi: 10.1371/journal.pone.0204669 (PMC6160104; doi:10.1371/journal.pone.0204669)

**VRP**

**HOL**

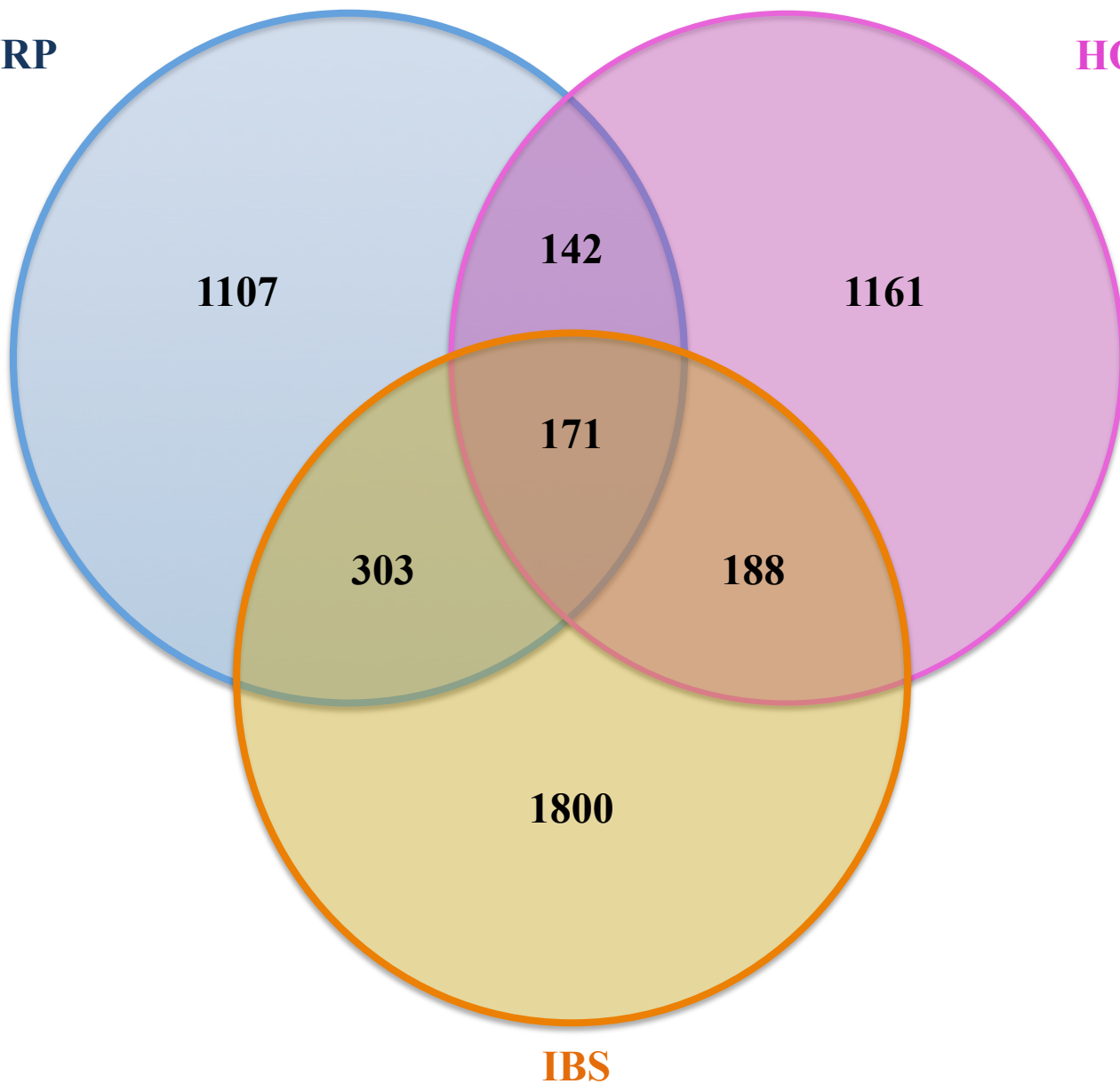

**IBS**

Supplement: S1 Fig — (PDF) [file pone.0204669.s001.pdf]
