# Supplementary material for: A copy number variant scan in the autochthonous Valdostana Red Pied cattle breed and comparison with specialized dairy populations
Source: PLoS One. 2018 Sep 27;13(9):e0204669. doi: 10.1371/journal.pone.0204669 (PMC6160104; doi:10.1371/journal.pone.0204669)
Supplement: S2 Table — (DOCX) [file pone.0204669.s003.docx]

Table S2. Descriptive statistics of VRP CNVRs classified according to state (gain, loss and complex) by chromosome (Chr).

| **Chr** | **Gain** | **Loss** | **Complex** | **Total** | **Coverage (%)** |
| --- | --- | --- | --- | --- | --- |
| 1 | 1349982 | 2259735 | 266665 | 3876382 | 6.52 |
| 2 | 720553 | 937300 | 200044 | 1857897 | 3.13 |
| 3 | 1578264 | 1001670 | 95246 | 2675180 | 4.5 |
| 4 | 906360 | 969635 | 111304 | 1987299 | 3.34 |
| 5 | 1496755 | 738116 | 1028981 | 3263852 | 5.49 |
| 6 | 1096467 | 1837569 | 202841 | 3136877 | 5.28 |
| 7 | 300420 | 995801 | 89277 | 1385498 | 2.33 |
| 8 | 1002345 | 1049434 | 46708 | 2098487 | 3.53 |
| 9 | 691913 | 1245259 | 62760 | 1999932 | 3.36 |
| 10 | 438819 | 1032251 | 1705600 | 3176670 | 5.34 |
| 11 | 486075 | 1488077 | 74432 | 2048584 | 3.45 |
| 12 | 1220992 | 1533642 | 6522241 | 9276875 | 15.61 |
| 13 | 838740 | 468882 | 0 | 1307622 | 2.2 |
| 14 | 425094 | 571938 | 18461 | 1015493 | 1.71 |
| 15 | 1086382 | 1032351 | 765341 | 2884074 | 4.85 |
| 16 | 896303 | 473656 | 380533 | 1750492 | 2.94 |
| 17 | 889917 | 890027 | 304084 | 2084028 | 3.51 |
| 18 | 894058 | 403655 | 0 | 1297713 | 2.18 |
| 19 | 434555 | 956299 | 232664 | 1623518 | 2.73 |
| 20 | 567541 | 880589 | 129189 | 1577319 | 2.65 |
| 21 | 442497 | 503195 | 98677 | 1044369 | 1.76 |
| 22 | 306104 | 261341 | 0 | 567445 | 0.95 |
| 23 | 598198 | 153223 | 794899 | 1546320 | 2.6 |
| 24 | 388987 | 169489 | 37330 | 595806 | 1 |
| 25 | 554332 | 50553 | 0 | 604885 | 1.02 |
| 26 | 138183 | 661169 | 21753 | 821105 | 1.38 |
| 27 | 660510 | 89094 | 0 | 749604 | 1.26 |
| 28 | 230050 | 1077040 | 0 | 1307090 | 2.2 |
| 29 | 1356214 | 488780 | 34689 | 1879683 | 3.16 |
| Total | 21996610 | 24219770 | 13223719 | 59440099 | 100 |
