# Supplementary material for: A copy number variant scan in the autochthonous Valdostana Red Pied cattle breed and comparison with specialized dairy populations
Source: PLoS One. 2018 Sep 27;13(9):e0204669. doi: 10.1371/journal.pone.0204669 (PMC6160104; doi:10.1371/journal.pone.0204669)
Supplement: S5 Table — (DOCX) [file pone.0204669.s006.docx]

Table S4. Descriptive statistic of CNVs and CNVRs count identified with PennCNV in Valdostana Red Pied (VRP), Holstein (HOL) and Italian Brown Swiss (IBS).

| **Breed** | **Animal count** | **CNV count** | **CNVR** | **Gain** | **Loss** | **Complex** | **Length (Mb)** |
| --- | --- | --- | --- | --- | --- | --- | --- |
| **VPR** | 108 | 6,784 | 1,723 | 832 | 812 | 79 | 59,44 |
| **HOL** | 124 | 12,070 | 1,662 | 394 | 1215 | 53 | 82,67 |
| **IBS** | 164 | 9,203 | 2,462 | 1196 | 1176 | 90 | 61,17 |
| **Total** | 396 | 28,057 | 5,847 | 2422 | 3203 | 222 | 203,28 |
